# Decoding leprosy: immune response, clinical manifestations and emerging diagnostic approaches

**DOI:** 10.1093/trstmh/traf058

**Published:** 2025-06-04

**Authors:** Héctor Serrano-Coll, Lina Restrepo-Rivera, Yicenia Cuadros-Urrego, Nora Cardona-Castro

**Affiliations:** Leprosy research group, Instituto Colombiano de Medicina Tropical-Universidad CES, Medellín 050001, Colombia; Leprosy research group, Instituto Colombiano de Medicina Tropical-Universidad CES, Medellín 050001, Colombia; Leprosy research group, Instituto Colombiano de Medicina Tropical-Universidad CES, Medellín 050001, Colombia; Leprosy research group, Instituto Colombiano de Medicina Tropical-Universidad CES, Medellín 050001, Colombia

**Keywords:** disability, early detection, immunology, leprosy, neglected disease, pathology

## Abstract

Leprosy is an infectious disease characterized by a broad immunological spectrum, which underlies its diverse clinical presentations and contributes to the physical and emotional disabilities experienced by patients with leprosy. Understanding the immunopathogenic mechanisms of this disease is essential for interpreting clinical manifestations and conventional diagnostic tests and developing diagnostic strategies for leprosy detection. This review aims to provide an overview of the immune response, clinical features and emerging diagnostic approaches in leprosy.

## Introduction

Leprosy, or Hansen's disease, is a chronic infection caused by *Mycobacterium leprae* and *Mycobacterium lepromatosis*.^1^ It primarily affects the skin and peripheral nerves.^2^ Leprosy is curable with appropriate treatment; however, if not detected early, it can lead to permanent disabilities in the eyes, hands and feet.^3^

Leprosy remains a significant public health concern, particularly in developing countries. According to the WHO, 182 815 new cases were reported worldwide in 2023.^2^ If left untreated, the disease can cause nerve damage, leading to sensory loss, deformities and physical disability.^4^^,^^5^ Additionally, affected individuals often experience stigma and discrimination, which hinder their social integration and access to healthcare services.^6^ Beyond physical impairments, leprosy also imposes substantial psychological and economic burdens, contributing to depression, anxiety and reduced quality of life.^7^^,^^8^

Although leprosy is not usually fatal, its consequences extend far beyond physical symptoms. The disease has historically been associated with social exclusion and emotional distress, with patients once described as ‘the living dead’, a perception dating back to the Middle Ages.^9^ Addressing this neglected disease requires not only improved diagnostic tools but also a stronger commitment to reducing its societal impact and ensuring better healthcare access for those affected.

Considering that leprosy transmission persists and detection is often delayed, leading to physical and social repercussions, we find it relevant to present this review, which aims to describe the immune response, clinical manifestations, conventional tests and emerging diagnostic approaches in leprosy detection.

## Host immune response to *M. leprae*

When *M. leprae* comes into contact with the host, it encounters the innate immune system as the first line of defense. This response involves the integrity of epithelial barriers, the activation of natural killer (NK) cells and the engagement of pattern recognition receptors, among which Toll-like receptors (TLRs) play a pivotal role.^10–12^ Activation of TLRs stimulates the production of cytokines and chemokines, which are crucial for initiating the adaptive cellular immune response against *M. leprae*.^13^

**Figure 1.**
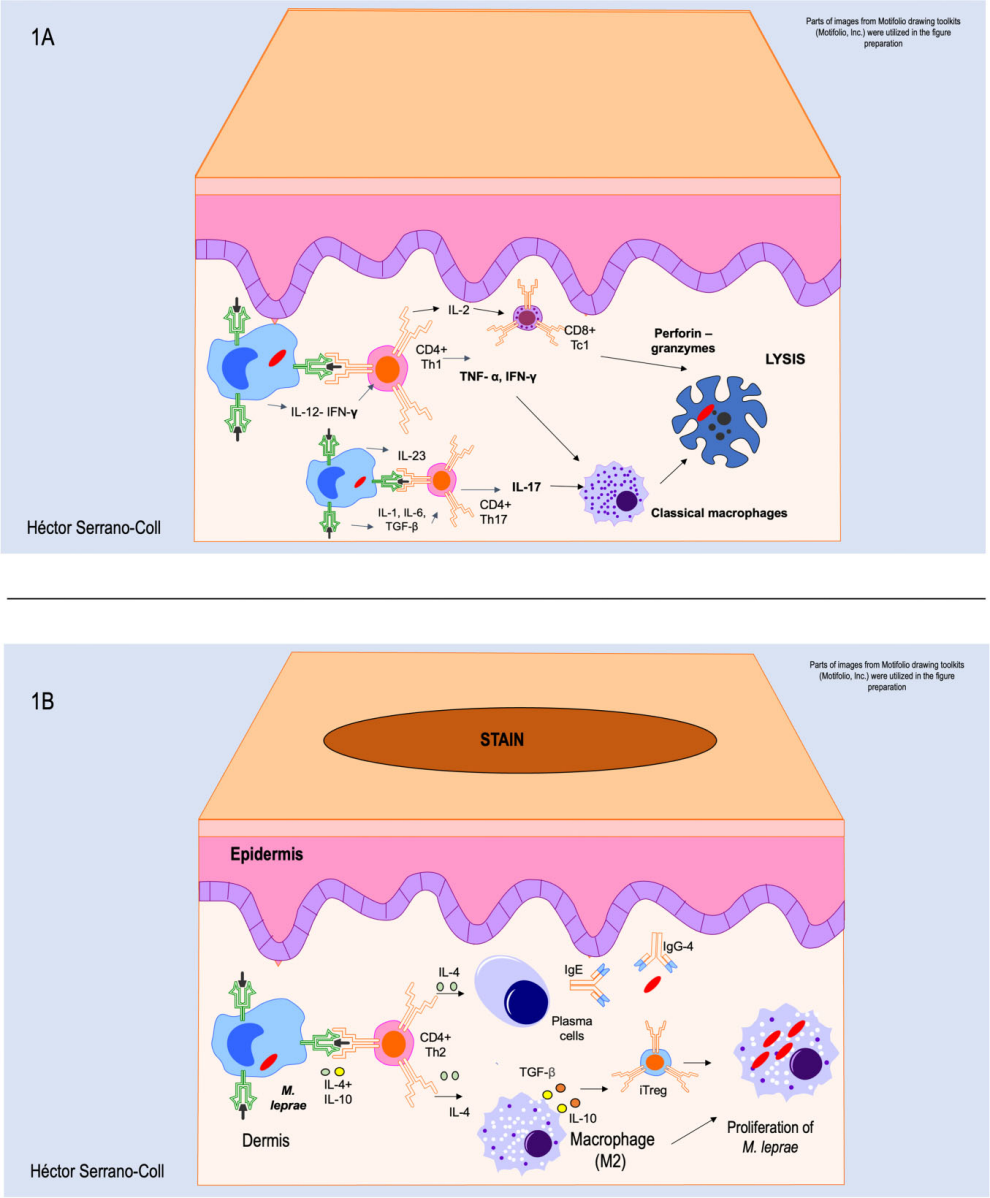
Host immune response to *M. leprae*. (A) The figure illustrates a hyperergic pattern against *M. leprae*, showing CD4+ T lymphocyte Th1 and Th17 activity, which promote the recruitment and differentiation into M1 macrophages as well as the cytotoxic activity of CD8+ lymphocytes targeting cells infected by this mycobacterium. (B) The figure shows the differentiation of T lymphocytes into a Th2 profile, resulting in an ineffective response to the infection, thereby favoring the growth and proliferation of *M. leprae*. iTreg: induced T regulatory lymphocytes.

The activation of the cellular immune response against *M. leprae* is initiated by antigen presentation, primarily carried out by dendritic cells, which present antigens to naive T lymphocytes via Major Histocompatibility Complex (MHC) molecules: MHC class II for CD4+ and MHC class I for CD8+ T cells.^14^ During this process, cytokines play a critical role, with IL-12 and IFN-γ driving the differentiation of CD4+ T cells into the Th1 effector subtype. At the same time, IL-6, TGF-β and IL-1 promote differentiation into the Th17 subtype. However, T lymphocytes can also acquire other effector profiles such as Th2, Th9 or even Th22, depending on the host's immune response to *M. leprae*.^15^ Besides, the IL-23/JAK-STAT signaling pathway plays a key role in sustaining the Th17 response.^16^ Once activated, CD4+ T cells adopt either a Th1 or Th17 profile; Th1 cells produce IL-2, which supports the proliferation of cytotoxic CD8+ T cells (Tc1).^5^^,^^17^ Additionally, TNF-α and IL-17, secreted by Th1 and Th17 CD4+ cells, contribute to the recruitment of inflammatory cells such as neutrophils and macrophages. Meanwhile, IFN-γ released by CD4+ Th1 cells enhances the immune response by promoting the classical activation of macrophages (M1), thereby increasing their microbicidal activity (Figure 1A).^18^ However, antigen presentation by dendritic cells can be impaired upon phagocytosis of *M. leprae*, as these cells express phenolic glycolipid I (PGL-I) in the cytoplasm and on their surface.^19^ PGL-I, a surface-expressed molecule involved in bacterial invasion, has been shown to downregulate MHC class I and II expression, thereby limiting antigen presentation and compromising T cell activation.^19^

On the other hand, the humoral response in the context of this infection has historically been underestimated and often considered inefficient against *M. leprae*. However, these effector patterns (Th1-Th17) are key in releasing IL-17 and IFN-γ by follicular T cells. This response promotes isotype switching in antibody-producing plasma cells toward IgG1 and IgG3 isotypes.^20^ These antibodies exhibit properties such as neutralizing activity, the ability to mediate antibody-dependent NK cell cytotoxicity and activation of the complement cascade through the classical pathway.^21^ Collectively, these immunological mechanisms significantly contribute to the elimination of *M. leprae*.

Hosts who develop leprosy can exhibit a wide spectrum of immune responses, ranging from anergic to hyperergic. An anergic response reflects weak immunity against mycobacteria, while a hyperergic response is characterized by stronger, although not fully effective, Th1 and Th17 activity.^22^^,^^23^ These responses help control the infection partially, yet fail to eliminate *M. leprae* entirely, resulting in chronic inflammation and the formation of granulomatous lesions in skin and peripheral nerves.^5^^,^^17^^,^^24^ The incomplete control despite Th1/Th17 activation suggests the existence of yet unidentified immunological mechanisms.^17^ Environmental factors such as malnutrition and overcrowding may further modulate host immunity, highlighting the need for continued research into these interactions.^25^^,^^26^ Moreover, negative regulators of the JAK-STAT pathway, such as SOCS1 (Suppressors of Cytokine Signaling-1),^27^ act as natural brakes on this signaling cascade and have been associated with leprosy susceptibility by partially or completely compromising Th17 activity, thereby favoring disease progression.^16^ Furthermore, immune responses may be suppressed by PGL-I, which inhibits the expression of inducible nitric oxide synthase (iNOS), essential for macrophage microbicidal activity. This inhibition occurs via complement receptor 3 (CR3) and disrupts TLR4 signaling by reducing TRIF levels, a key adaptor for iNOS induction.^28^

On the other hand, individuals who exhibit an anergic immune response to the infection will develop a generalized form of the disease known as lepromatous leprosy (LL). Here, alterations in TLR-mediated signaling induce the expression of IL-10 and IL-4 while inhibiting IL-12, which promotes the differentiation of CD4+ T cells toward a Th2 effector pattern.^17^^,^^24^ The release of IL-4 by Th2 cells promotes the differentiation of macrophages into an alternatively activated phenotype (M2), characterized by the release of anti-inflammatory cytokines such as IL-10 and TGF-β,^29^ which creates a permissive environment for the proliferation of *M. leprae* within skin and Schwann cells.^13^^,^^18^ Furthermore, the Th2 immune response induces isotype switching in plasma cells toward IgE and IgG4.^18^ These isotypes exhibit limited capacity for opsonization and neutralization,^18^^,^^30^ which contributes to a suboptimal humoral response against *M. leprae* and facilitates the proliferation of the mycobacterium in host tissues^5^ (Figure 1B). Moreover, in LL, several miRNAs such as hsa-miR-16–5p, miR-20a-5p and miR-106b-5p may be deregulated and contribute to an impaired immune response. These miRNAs suppress key genes such as IFNG, SMAD7 and CHUK, reducing macrophage activation, the production of pro-inflammatory cytokines and promoting an immunosuppressive profile dominated by TGF-β and IL-10.^31^

An important consequence of M2 macrophage polarization in leprosy is the establishment of an anti-inflammatory environment, driven by the production of TGF-β and IL-10, along with the expression of markers such as CD163.^17^ In in vitro studies, monocyte-derived macrophages exposed to live *M. leprae* acquired an M2 profile, with decreased expression of IL-1β, IL-6, TNF-α and MHC class II molecules, and increased IL-10 production.^32^ When co-cultured with naive T cells, these macrophages promoted a regulatory response, favoring the differentiation of regulatory T (Treg) cells with elevated FoxP3 and IL-10 expression, and reduced IFN-γ production and CD8⁺ T cell-mediated cytotoxicity.^32^ In this context, it is likely that these regulatory subsets correspond to induced regulatory T cells, such as Tr1 cell characterized by IL-10 production and Th3 cells driven by TGF-β,^33^ which may contribute to the establishment of an immunosuppressive microenvironment that limits cellular immune responses and facilitates bacillary persistence.

## Clinical features and association with conventional tests in leprosy

The clinical manifestations of leprosy depend on the host's cellular immune response to *M. leprae*. These manifestations are preceded by long incubation periods, which range from 6 mo to up to 20 y.^10^ This prolonged incubation is attributed to the mycobacterium's slow proliferation, low antigenicity and metabolic limitations.^10^ Clinically, leprosy has been classified into stable and unstable spectrums. The stable forms include tuberculoid leprosy (TL) and LL, as their clinical, histopathological, bacteriological and immunological characteristics remain constant over time. By contrast, unstable forms include indeterminate leprosy (IL), which can evolve into tuberculoid, borderline or lepromatous forms as the disease progresses. Similarly, borderline forms are also considered unstable, as some cases may shift toward either the tuberculoid or lepromatous poles of the spectrum.^34^ The main clinical features are described below.

### Indeterminate leprosy

**Figure 2.**
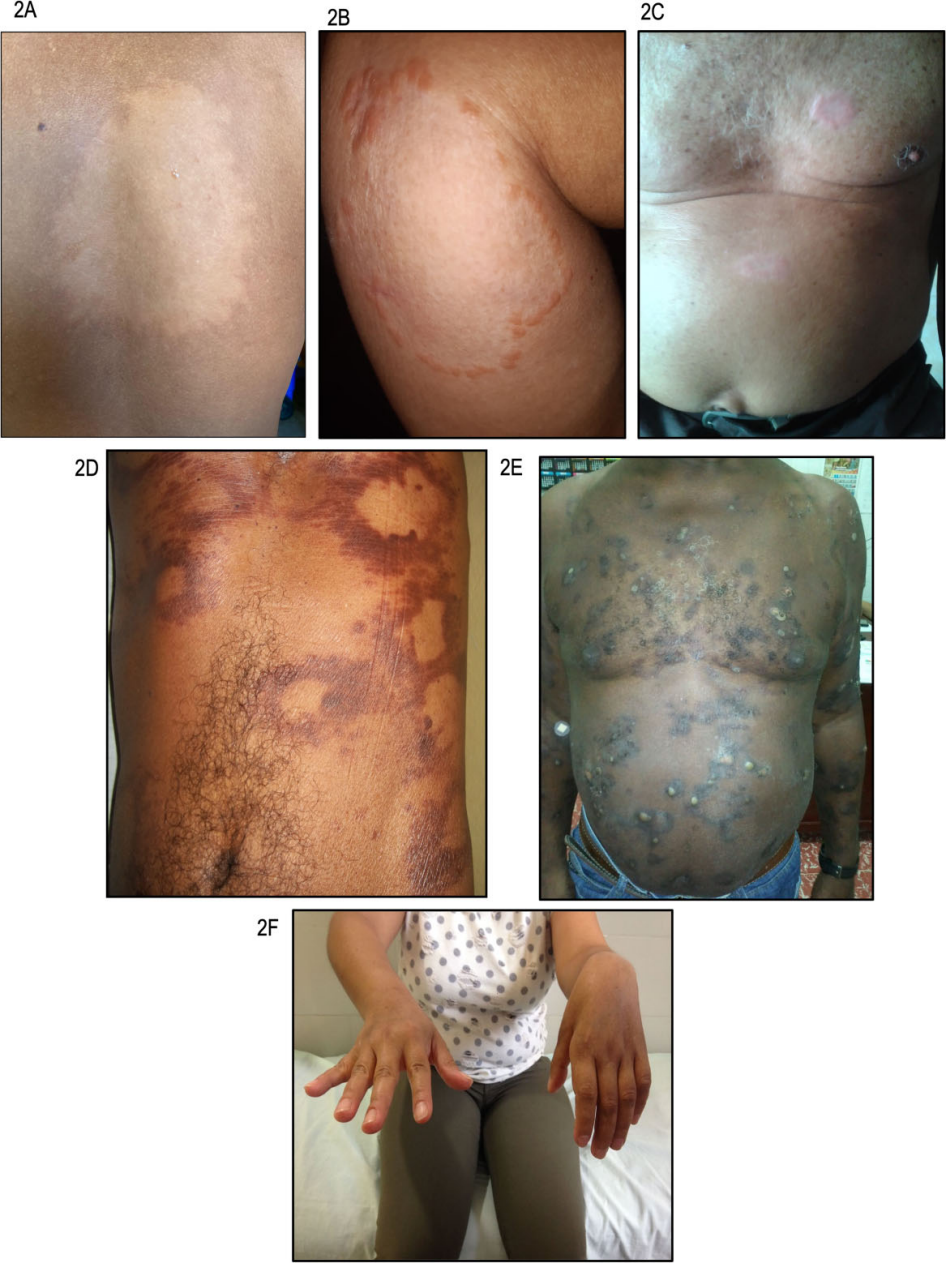
Clinical characteristics of leprosy. (A) Indeterminate leprosy; the image shows a single hypopigmented lesion with poorly defined borders. (B) Tuberculoid leprosy; the image depicts a single granulomatous-bordered lesion on the lower limb. (C) Lepromatous leprosy; the image demonstrates countless lesions on the chest and abdomen, accompanied by nodules and plaques. (D) Borderline tuberculoid leprosy; the image shows multiple lesions with granulomatous and erythematous borders. (E) Borderline lepromatous leprosy; the image illustrates numerous borderline lesions on the chest and abdomen. (F) Neural leprosy; the image reveals left hand drop with motor involvement of the radial nerve.

IL is considered the initial form of this disease. Clinically, it is characterized by a few skin lesions, typically hypopigmented, flat and with poorly defined borders (Figure 2A).^34^ The most common locations include the face, trunk and upper limbs. From a neurological perspective, these lesions are associated with microneuropathies, evidenced by hypoesthesia—most commonly to temperature—in circumscribed areas within the lesions, without involvement of peripheral nerve trunks.^35^

Immunologically, this initial form does not exhibit a clear Th1+Th17 effector pattern or a Th2 response. It explains the incipient and undefined nature of the skin lesions and the minimal neural damage observed in these patients.^36^

**Figure 3.**
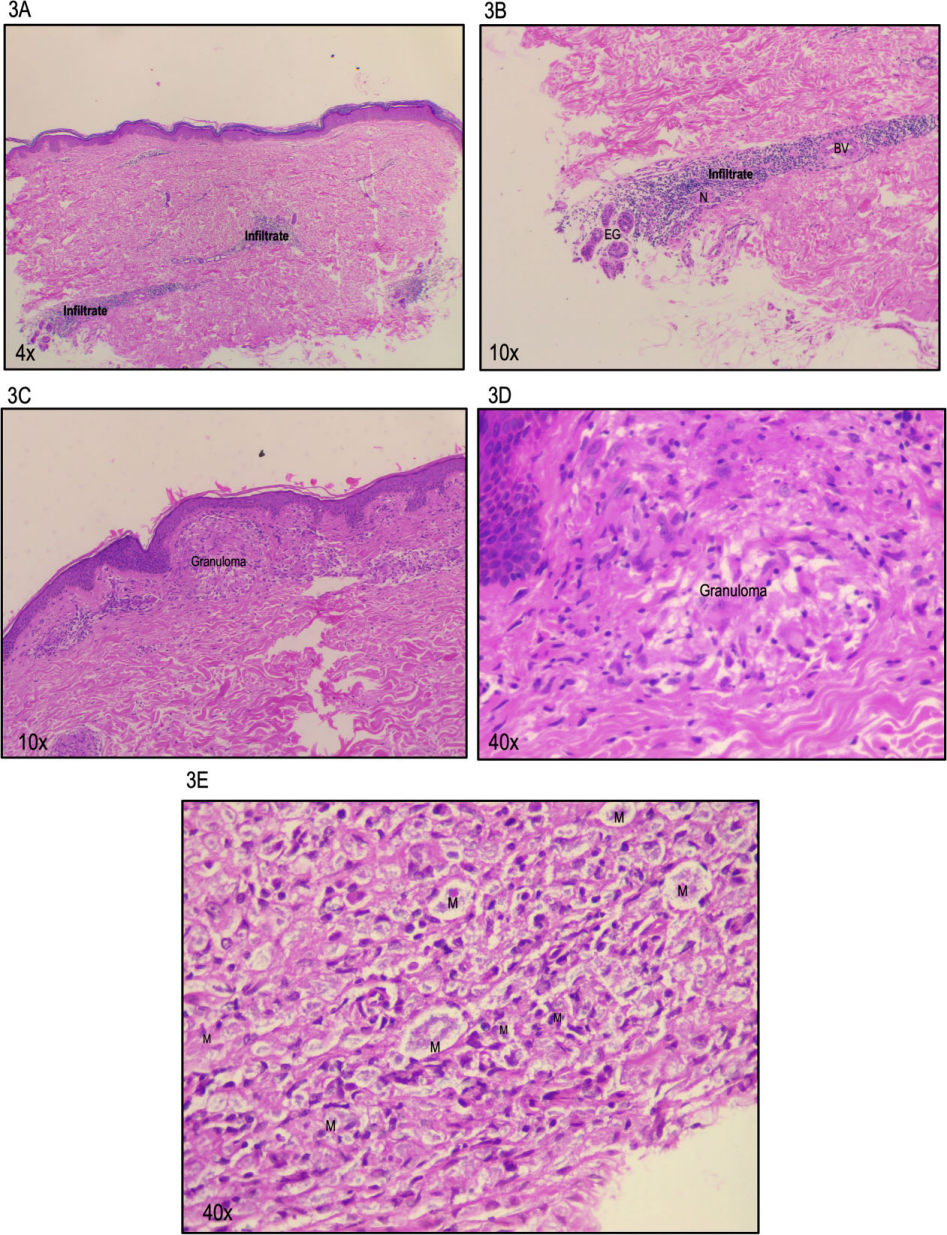
Histopathological features of leprosy. (A,B) These micrographs of indeterminate leprosy show a lymphoplasmacytic infiltrate surrounding eccrine glands (EG), nerve bundles (N) and blood vessels (BV). (C,D) Micrographs of tuberculoid leprosy display a well-formed granuloma beneath the epidermis. (E) Micrograph of lepromatous leprosy reveals numerous foamy macrophages (M).

Regarding diagnosis, bacilloscopy is often negative due to the low bacillary load, limiting its utility.^37^ While molecular tests can provide valuable information, the low bacterial load in this early form of the disease poses challenges to amplifying the pathogen's genetic material.^37^ On the other hand, histopathology can be helpful through indirect findings, such as the presence of a lymphoplasmacytic infiltrate surrounding dermal nerve fibers or cutaneous appendages (Figure 3A,B).^34^ Table 1 describe the clinical, immunological and paraclinical features of the leprosy spectrum.

**Table 1.** Clinical, immunological and paraclinical features of leprosy

**Leprosy spectrum**	**Clinical features**	**Immune pattern**	**Bacilloscopy**	**Histopathological features**	**References**
Indeterminate leprosy	<5 hypopigmented, flat skin lesions with poorly defined borders	Th1-Th17 or Th2	Negative	LPI surrounding dermal nerve fibers or cutaneous appendages	^34–36^
TL	<5 infiltrated skin lesions, granulomatous borders, dryness, alopecia and anesthesia	Th1-Th17	Negative	Granulomas composed of epithelioid cells and FBGC	^38^ ^,^ ^39^
BT	5–20 skin lesions, granulomatous in appearance, resembling TL Satellite lesions observed around larger ones	Th1-Th17	Depends on the number of skin lesions: <5 lesions negative; >5 lesions could be positive (1–2+)	BT has histopathological similarities with TL: Inflammatory infiltrates may focally erode the epidermis Granulomas composed of epithelioid cells and FBGC are present in the dermis Differences with BL: Presence of a Grenz zone or Unna band F-F staining may also be positive	^43^ ^,^ ^45^ ^,^ ^47^
Borderline borderline leprosy	Multiple borderline-like skin lesions	Th2, with some Th1/Th17 activity	Positive (3–4+)	Granulomas mainly composed of epithelioid cells, with rare MGC F-F staining is typically positive	^13^ ^,^ ^21^ ^,^ ^37^
BL	Multiple borderline-like skin lesions	Th2	Positive (4–5+)	Dermal granulomas of vacuolated histiocytes, abundant lymphocytes Nerve involvement shows ‘onion-skin’ perineurium with lymphocytes F-F staining highlights bacilli	^37^ ^,^ ^44^ ^,^ ^50^
Lepromatous leprosy	Multiple macules and papules, progressively evolving into nodules and plaques	Th2	Positive (4–6+)	Granulomas composed of foamy histiocytes, vacuolated MGC F-F staining highlights bacilli	^44^ ^,^ ^52^
Neural leprosy	Absence of skin lesions, with the primary involvement of peripheral nerve trunks	Th1-Th17 or Th2	Negative	Diagnostic criteria for neural leprosy include: Fibrosis or chronic lymphohistiocytic inflammation observed in peripheral nerve biopsy PCR for *M. leprae* could be a useful adjunct to improve diagnostic accuracy	^55–57^

BL: borderline lepromatous leprosy; BT: borderline tuberculoid leprosy; FBGC: foreign body-type giant cells; F-F: Fite-Faraco; LPI: lymphoplasmacytic infiltrate; MGC: multinucleated giant cells; TL: tuberculoid leprosy.

Here, it is important to mention that PCR can improve the detection of *M. leprae* in IL, with a positivity rate of 75.61% for multiplex PCR in the indeterminate form compared with 58.54% for RLEP PCR. This enhances early diagnosis in cases with a low bacillary load.^38^

### Tuberculoid leprosy

The clinical form of TL is characterized by a few infiltrated lesions, which are well demarcated by a granulomatous border, sparse in number (<5 lesions) and asymmetrically distributed as xerotic alopecic plaques. These lesions show significant sensory impairment (Figure 2B).^39^

From an immunological perspective, TL represents a hyperergic form, with a marked imbalance favoring heightened activity of CD4+ T cells with an effector Th1 and Th17 profile.^17^^,^^24^ These factors promote intense inflammation, contributing to the formation of perilesional granulomas, demyelination and nerve fiber damage.^5^ The characteristic Th1 and Th17 responses in this condition explain the absence of bacilli in the bacilloscopy of affected patients.^34^ On the other hand, histopathologically, multiple granulomas composed of epithelioid cells and foreign body-type giant cells can be observed (Figure 3C,D).^40^ The overactivity of Th1- and Th17-mediated immune responses contributes to nerve damage, as evidenced by the fragmentation or disappearance of nerves.^5^ Consequently, the histopathological diagnosis of this condition is primarily based on identifying these indirect findings (Table 1).

### Borderline leprosy

Borderline leprosy represents an intermediate clinical spectrum between TL and LL. For better understanding, borderline leprosy is subdivided into three clinical forms, namely, borderline tuberculoid leprosy (BT), borderline borderline leprosy (BB) and borderline lepromatous leprosy (BL) (Table 1).^41^ Additionally, it is important to note that this clinical form is often associated with type IV hypersensitivity reactions, known as type I leprosy reactions (upgrading or downgrading). These reactions are characterized by an immune response driven by CD4+ T cells, with a predominant Th1 and Th17 effector pattern.^42^ This immune activation can lead to an immune response miming a shift in the clinical-histological Ridley–Jopling classification, as patients transition from the borderline spectrum toward the tuberculoid form of leprosy.^43^

#### Borderline tuberculoid leprosy

BT lesions appear as granulomatous plaques, similar to the tuberculoid form, but they differ in being more numerous, with less regular borders, and are less dry and less alopecic (Figure 2C). Additionally, satellite lesions can be observed around larger lesions.^13^^,^^34^ Significant sensory impairment is present, and multiple nerve trunks may be thickened.^44^

This clinical form is characterized by a Th1 and Th17-type immune response, although less prominent than that observed in TL.^24^^,^^45^ However, this partially hyperergic immune response is responsible for both the granulomatous characteristics of the cutaneous lesions and the peripheral nerve damage experienced by patients.^36^

From a diagnostic perspective, bacilloscopy in these cases can yield either positive or negative results,^46^ underscoring the importance of classifying patients based on the number of skin lesions. The disease is considered multibacillary (MB) when the patient presents with >5 lesions and paucibacillary (PB) when there are ≤5.^47^ Besides, histopathology in this clinical presentation shares similarities with TL.^48^ However, it presents specific features, such as an epidermis that may be focally eroded by inflammatory infiltrates.^36^ Additionally, the Unna band or Grenz zone is frequently observed, which is a layer of connective tissue beneath and parallel to the epidermis that separates it from the inflammatory infiltrate.^13^ Fite-Faraco staining could be positive in this clinical form.^34^

#### Borderline borderline leprosy

BB is an intermediate clinical form of borderline leprosy that is characterized by multiple borderline-like skin lesions. These lesions are typically described as having a rounded center, with the inner margin being more sharply defined than the outer margin, resembling Swiss cheese.^20^

From an immunological perspective, BB represents a clinical form characterized by an immune response skewed toward a Th2 pattern, although less pronounced compared with other forms within the lepromatous spectrum.^13^ Therefore, this Th2 immunological pattern contributes to the presence of multiple skin lesions in affected patients, and bacilloscopy frequently yields positive results.^37^ Histopathological evaluation reveals granulomas predominantly composed of epithelioid cells; however, multinucleated giant cells are rarely observed.^13^ Additionally, Fite-Faraco staining is typically positive.^13^

#### Borderline lepromatous leprosy

BL in its clinical form may be mistaken for LL, but can be distinguished by the presence of borderline-like lesions and greater neurological involvement (Figure 2D).^13^^,^^37^

BL falls within the Th2-dominated immune spectrum, although this anergic immune response is less severe than fully lepromatous leprosy.^17^ This immune response leads to multiple, widespread skin lesions.^34^ However, this clinical form lacks the characteristic facial, otorhinolaryngological and testicular involvement observed in LL.^49^ Besides, BL usually shows a positive bacilloscopy with a high bacillary index.^44^ Histopathological analysis reveals a thin epidermis and an inflammatory infiltrate in the dermis, predominantly consisting of granulomas formed mainly by foamy, vacuolated histiocytes or macrophages, along with abundant lymphocytes.^50^ This infiltrate is often diffuse, irregular, nodular, perivascular and present around adnexal structures.^50^ Nerve involvement shows ‘onion-skin’ perineurium with lymphocytes forming cuffs around nerve bundles.^51^ Fite-Faraco staining is positive, revealing bacilli within macrophages, Schwann and endothelial cells (Table 1).^48^

### Lepromatous leprosy

The clinical form of LL is characterized by macules, papules, nodules and plaques with a symmetrical, bilateral and diffuse distribution (Figure 2E).^44^ Besides, LL is accompanied by leonine facies, frontal infiltration, madarosis, loss of eyelashes, involvement of the nasal septum, testicular atrophy, impotence, infertility and gynecomastia.^44^ These clinical findings are related to the Th2 immunological pattern that creates a favorable environment for the proliferation and dissemination of *M. leprae* across multiple body structures.^52^ Furthermore, patients with LL frequently develop type III hypersensitivity reactions mediated by immune complexes, known as type II leprosy reactions or erythema nodosum leprosum.^24^ The deposition of these immune complexes in tissues triggers an inflammatory response that can become systemic, leading to inflammation in the skin, peripheral nerves, kidneys, liver and other organs.^43^

On the other hand, the bacilloscopy in LL is considered the laboratory gold standard, playing a critical role in confirming the disease.^44^ Patients with LL typically present with high bacillary indices, ranging from 4+ to 6+ on the Ridley international scale. Additionally, due to the significant bacillary burden, molecular tests are often positive.^44^ The histopathological findings include an atrophic or thinned epidermis, with the constant presence of Unna's band or Grenz zone.^40^^,^^53^ The inflammatory infiltrate consists of granulomas formed by foamy histiocytes, vacuolated cells, multinucleated giant cells with vacuoles, and scant lymphocytes and plasma cells; this infiltrate may extend into the deep dermis and subcutaneous fat (Figure 3E).^40^ Besides, the Fite-Faraco staining is positive, revealing bacilli within macrophages, Schwann and endothelial cells (Table 1).^40^^,^^54^

### Neural leprosy

Neural leprosy (NL) is a peripheral neuropathy characterized by the absence of skin lesions, with the primary involvement of peripheral nerve trunks, leading to sensory alterations in the innervated areas (Figure 2F).^55^

The pathophysiology of NL remains poorly understood. However, research by Pitta et al.^56^ has demonstrated that cytokines such as TNF-α and IL-17, associated with Th1 and Th17 patterns, contribute to demyelination and axonal degeneration. Additionally, this study revealed that IL-10, linked to the Th2 pattern, is involved in inducing axonal damage and silent neuritis. These findings highlight NL as a unique form of leprosy.

On the other hand, NL is characterized by a negative slit skin smear bacilloscopy.^57^ Besides, the histopathological findings reveal fibrosis or chronic lymphohistiocytic inflammation within the peripheral nerve biopsy.^58^ In such cases, molecular testing can help detect *M. leprae*, although results can sometimes be ambiguous.^57^ Nonetheless, PCR significantly enhances the sensitivity for detecting *M. leprae*, particularly in probable NL cases where bacilli cannot be demonstrated through histopathology (Table 1).^59^

## Advances in diagnostic methods in leprosy detection

Despite advances in diagnostic methods for infectious diseases, the disparity in the availability of routine diagnostic tools for leprosy in endemic areas—compared with those for TB—is both striking and concerning. One of the most significant challenges in leprosy diagnosis is the lack of a reliable screening test capable of detecting the disease in its early stages, particularly in IL and TL, which fall within the PB spectrum. This limitation underscores the importance of establishing appropriate sensitivity and specificity criteria to improve detection accuracy. Addressing these challenges requires the development of diagnostic tools that can effectively identify *M. leprae* across different endemic contexts, ensuring timely intervention and reducing transmission rates.^60^

Molecular tests such as PCR allow the detection of specific nucleic acids of *M. leprae*,^61^ but their availability remains limited to specialized research laboratories and a few public health centers, making routine use challenging. By contrast, TB diagnosis has significantly advanced with GeneXpert technology,^62^ an automated and user-friendly system that not only detects *Mycobacterium tuberculosis* but also identifies rifampicin resistance mutations in the *rpoB* gene, improving early detection and treatment in endemic areas.^63^ However, in the case of leprosy, the emergence of multidrug-resistant strains presents an additional challenge, highlighting the need for diagnostic tools focused on their detection.^64^ In this context, the GenoType LepraeDR assay has demonstrated 100% concordance with PCR sequencing and mouse susceptibility models, enabling the identification of mutations in the *rpoB, folP1* and *gyrA* genes, responsible for resistance to rifampicin, dapsone and fluoroquinolones, respectively.^65^ Additionally, qPCR-HRM analysis has proven to be a rapid and reliable tool for large-scale resistance surveillance, showing 100% concordance with direct DNA sequencing and a sensitivity of 98.7% in MB cases and 63.3% in PB cases.^66^ However, to minimize the risk of false positives, it is recommended to confirm the presence of *M. leprae* using qPCR-RLEP before performing susceptibility analysis.^66^ These advances establish molecular testing not only as a key tool for detecting *M. leprae* but also as a fundamental resource for antibiotic resistance surveillance and the optimization of therapeutic strategies in leprosy.

Alternative methods for evaluating cellular immune response have been developed. In TB diagnostics, the Interferon-Gamma Release Assay measures specific gamma interferon levels in response to *M. tuberculosis* antigens, providing a highly specific alternative to purified protein derivative, although it remains costly and is primarily available in third-level laboratories.^67^ By contrast, leprosy lacks a widely available commercial test to assess cellular immunity, highlighting the need for further research and development in diagnostic tools for neglected tropical diseases. As lepromin testing becomes obsolete, advances in immunological and molecular diagnostics are crucial for improving early detection and management of leprosy. Novel biomarkers and immunological assays could enhance leprosy screening, particularly in endemic regions with limited resources.

Serological tests, such as NDO-LID and PGL-I, have demonstrated utility in leprosy screening, particularly in endemic regions. Reported sensitivities range from 34.0% and 32.0% in PB leprosy cases to 73.6% and 81.0% in MB cases in Brazil.^68^ In Colombia, sensitivities of 78.0% and 89.6% were observed in MB cases, whereas values of 6.9% and 20.3% were reported for PB cases, respectively.^69^ However, their diagnostic performance in PB cases remains limited, prompting the investigation of molecular tools such as *M. leprae* detection via RLEP qPCR, which has exhibited high sensitivity, confirming 86% of cases in field studies in Brazil.^70^ Additionally, antibody titers against PGL-I, LID-1 and NDO-LID decrease significantly after 6 and 12 mo of treatment, suggesting their potential use in monitoring therapeutic response. Nevertheless, although elevated anti-PGL-1 levels have been associated with an increased risk of disease development in household contacts, their utility in detecting preclinical cases remains uncertain, as more than one-half of seropositive individuals never progress to active disease.^71^

On the other hand, innovative diagnostic tools such as the RISK4LEP biomarker (*MT-ND2, REX1BD, TPGS1* and *UBC*) have shown potential for the early detection of leprosy. Changes in the expression of this genetic signature have been associated with an increased risk of developing the disease and can predict the onset of skin lesions 4–61 mo before they become clinically evident.^72^ Additionally, the newly developed multi-biomarker test, a lateral flow assay designed to enhance leprosy diagnostics by detecting six host serum proteins associated with immunity against *M. leprae*, could play a crucial role in quantitatively differentiating MB and PB leprosy cases from uninfected individuals in both high- and low-endemic areas.^73^ Furthermore, the transcriptomic marker *OCT6* has shown altered expression patterns in leprosy, with decreased expression in molecular studies suggesting neural damage in patients with leprosy.^74^ Therefore, this gene could serve as an auxiliary marker for leprosy. Additionally, given that *M. leprae* cannot be cultured in axenic media, a molecular viability assay based on the expression of *hsp18* and *esxA* has been developed. This assay has demonstrated a strong correlation with bacillary load and treatment duration, highlighting its potential for monitoring therapeutic efficacy, detecting relapses and evaluating new drugs in clinical trials.^75^

Currently, machine learning models are emerging as promising tools to enhance leprosy diagnosis. A recent study demonstrated that artificial neural networks can improve the histopathological evaluation of patients with leprosy, providing greater accuracy and efficiency in diagnosis.^76^

## Conclusions and future directions

Leprosy is a disease characterized by a broad spectrum of clinical manifestations, reflecting its complex and multifaceted immunopathogenesis, much of which remains poorly understood. This inherent complexity significantly hinders early diagnosis, contributing to delayed treatment, the progression of disabilities and reduced quality of life for those affected.

One of the most pressing challenges in combating leprosy is developing diagnostic tools to detect the disease early across its diverse clinical spectrum. Achieving this would have a transformative impact on global leprosy control efforts. However, this objective necessitates a substantial commitment to research focused on unraveling the pathophysiological mechanisms underlying each clinical form of the disease. Such insights are essential for designing diagnostic strategies that address the full range of clinical presentations, ultimately advancing efforts toward properly eliminating leprosy. In turn, this would markedly reduce the burden of disabilities and help alleviate the stigma associated with the disease, improving the lives of those affected.

## Data Availability

Not applicable.
